# Gsdma3 regulates hair follicle differentiation via Wnt5a-mediated non-canonical Wnt signaling pathway

**DOI:** 10.18632/oncotarget.22212

**Published:** 2017-10-31

**Authors:** Long He, Mingxing Lei, Yizhan Xing, Yuhong Li, Chunyan Hu, Peixing Chen, Xiaohua Lian, Tian Yang, Wanqian Liu, Li Yang

**Affiliations:** ^1^ “111” Project Laboratory of Biomechanics and Tissue Repair & Key Laboratory of Biorheological Science and Technology of Ministry of Education, College of Bioengineering, Chongqing University, Chongqing 400044, China; ^2^ Integrative Stem Cell Center, China Medical University Hospital, China Medical University, Taichung 40402, Taiwan; ^3^ Institute of New Drug Development, College of Biopharmaceutical and Food Sciences, China Medical University, Taichung 40402, Taiwan; ^4^ Department of Cell Biology, Third Military Medical University, Chongqing 400038, China

**Keywords:** non-canonical Wnt signaling pathway, Gsdma3, hair follicle, epithelial-mesenchymal interaction, differentiation

## Abstract

Hair follicle is a mini-organ that consists of complex but well-organized structures, which are differentiated from hair follicle progenitor or stem cells. How non-canonical Wnt signaling pathway is involved in regulating hair follicle differentiation remains elusive. Here we showed that *Wnt5a* regulates hair follicle differentiation through an epithelial-mesenchymal interaction mechanism in mice. We first observed that *Wnt5a* is expressed in the epithelial and dermal papilla cells during hair follicle development and growth. For the upstream of Wnt5a, RT-PCR and immunohistochemistry staining showed that *Wnt5a* expression is significantly decreased in the *Gsdma3*-mutant mice *in vivo*. Overexpression of *Gsdma3* results in a significantly increased expression of *Wnt5a* in the cultured epidermal cells *in vitro*. We also checked the downstream factors of Wnt5a by adenovirus-mediated overexpression of *Wnt5a* to the dermal papilla cells isolated from the mouse whisker. We found that overexpression of *Wnt5a* suppresses canonical Wnt signaling pathway effectors such as β-catenin and Lef1. In addition, genes involved in maintaining cell quiescent state are also significantly decreased in their expression to the DP cells which were treated by Wnt5a. Our study indicates that Wnt5a mediates epithelia-expressed Gsdma3 to influence DP cell behaviors, which in turn regulate hair follicle epithelia differentiation in mice.

## INTRODUCTION

Ectodermal organs consist of two important cellular partitions including epithelium and mesenchyme, which develop from different germ layers during early developmental stages but influence each other by direct cell-cell interactions or paracrine factors such as growth factors and extracellular matrix [1]. Epithelial-mesenchymal interactions function virtually in every step of ectodermal organogenesis. Hair follicle positions as a consummate and highly tractable model to study epithelial-mesenchymal interactions. It includes both epithelial tissues and a group of specified mesenchymal cells known as dermal papilla (DP) [2]. Epithelial-mesenchymal interactions play roles in nearly all development, growth and regeneration stages of hair follicle, to coordinate the formation of complex cellular components and structures of the follicle. Reciprocal signaling between the epidermal progenitor cells and the specialized dermal cells leads to the formation of hair placode and dermal condensate, which together grow downward to form a follicle in the dermis [2, 3]. After development, hair follicle undergoes cyclic growth consisting of anagen, catagen and telogen phases [4–6].

Several pathways have been identified to be essential for hair follicle development. Among them, Wnt signaling emerges as one of the earliest and most-important pathways [7]. Nineteen Wnt ligands have been discovered in human and mouse tissues. These Wnt ligands are classified to mediate canonical Wnt signaling pathway or non-canonical Wnt signaling pathway, both of which exert extensive physiological roles in governing cellular behaviors through an autocrine or paracrine mechanism during hair follicle development. For example, Wnt signaling pathway functions on specifying hair stem cell fate through a Wnt-SHH antagonism [8]. The canonical Wnt signaling pathway which utilizes β-catenin to relay the signal has been more studied in hair biology [7, 9–12]. Non-canonical Wnt signaling pathways are mainly categorized into three pathways, including the Wnt-cGMP/Ca2+ pathway, Wnt/planar cell polarity (PCP) pathway, and the Wnt-receptor tyrosine kinase-like orphan receptor 2 pathway [13]. Little is known how non-canonical Wnt signaling is mediated via epithelial-mesenchymal interactions to commit hair follicle development.

*Wnt5a* plays widely roles in regulating cellular behaviors in many tissues. Combined with Ror-Dishevelled, *Wnt5a* constitutes a core non-canonical Wnt pathway to regulate tissue morphogenesis during embryonic development [14]. Wnt5a is able to enhance mammary epithelial cell growth through receptor-like tyrosine kinase (RYK) [15]. It maintains hematopoietic stem cells in a quiescence state through suppressing *Wnt3a*-mediated canonical Wnt signaling pathway [16]. Mutation of *Wnt5a* results in increased angiogenesis through non-canonical Wnt signaling pathway [17]. In the hair follicle, *Wnt5a* is expressed in both epithelia and mesenchyme during hair development and cycling [18]. While *Wnt5a*-null mice display no apparent defect in embryonic hair follicle morphogenesis [19], overexpression of *Wnt5a* causes a delayed hair regeneration through inhibiting the canonical Wnt signaling pathway [13, 20, 21]. Recent study shows that adenovirus-mediated overexpression of *Wnt5a* in the cultured whisker results in a shortened hair shaft in length [22]. Mesenchymal *Wnt5a* mediates epithelial Notch/CSL signaling to control keratinocytes differentiation in the hair follicle [23]. These studies indicate that *Wnt5a* is largely associated with maintenance of stem cell fate, and inhibits hair follicle differentiation. Whereas the mechanism that how *Wnt5a* regulates cell differentiation in the hair follicle remains largely unknown.

*GasderminA3* (*Gsdma3*) as a key member of *gasdermin* gene family is specifically expressed in the epithelial tissues, including mouse skin keratinocytes [24]. *Gsdma3*-mutant mice display disorders in hair cycling, with hair loss during postnatal life [25–27]. We recently uncovered that disorders during catagen-telogen transition in *Gsdma3*-mutant mice is attributed to increased canonical Wnt signaling in the epithelia of hair follicle [24]. The increased expression of *Wnt10b* promotes cell proliferation in the epithelial strand, resulting in a failure of telogen entry of hair follicles in Gsdma3-mutant mice. Gsdma3 also regulates differentiation of epithelial tissues in the skin, such as the hair follicles [27]. Mutation of *Gsdma3* causes a loss of interlocking structures, leading to the defects in hair shaft anchoring to the follicle. However, the molecular mechanism that how Gsdma3 regulates hair follicle differentiation remains unclear.

In the present study, we first examined the expression of *Wnt5a* and *Gsdma3* in hair follicle in mice. To check the impact of loss of function of *Gsdma3* on *Wnt5a* expression, we compared *Wnt5a* expression in the hair follicle of *Gsdma3*-mutant mice and wild type mice. We also investigated the effect of gain of function of *Gsdma3* on *Wnt5a* expression in epidermal cells. By applying adenovirus-mediated overexpression of *Wnt5a* to the isolated dermal papilla cells, we evaluated the downstream targets of *Wnt5a* that mediate the non-canonical Wnt signaling in the DP cells. Our study demonstrates that epithelial *Gsdma3* positively regulates expression of *Wnt5a*, which influences the DP cell behaviors that further impact epithelial cell differentiation in an epithelial-mesenchymal interaction mechanism.

## RESULTS

### Wnt5a expression during hair development

**Figure 1:**
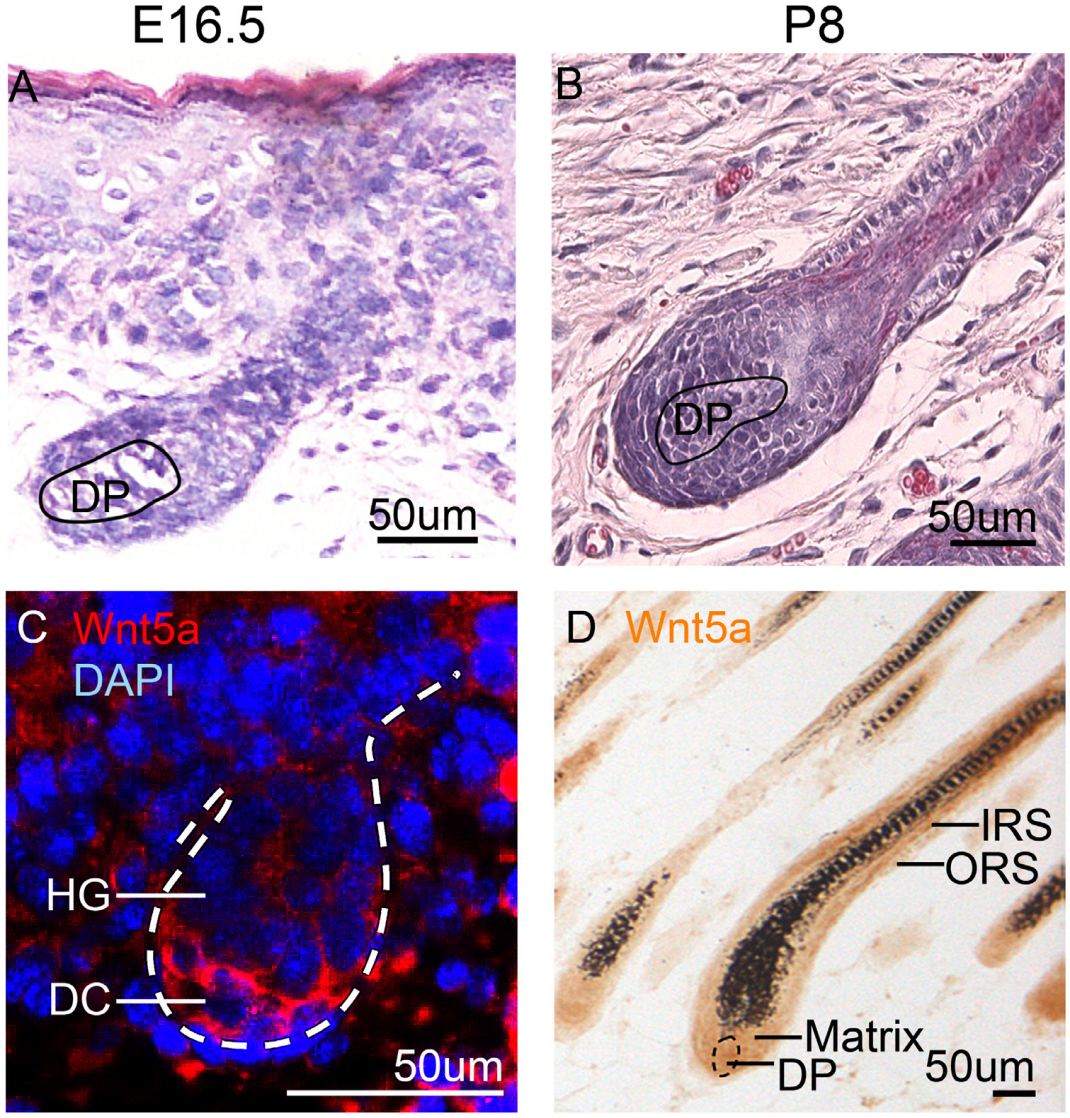
Wnt5a expression during hair development and growth **(A)** H&E staining shows a developing hair follicle at E16.5. **(B)** H&E staining shows a developed hair follicle in full anagen at P8. **(C)** Immunofluorescent staining shows that Wnt5a is expressed in the mesenchymal cells of skin, particularly in the DP at E16.5. **(D)** Immunohistochemistry staining shows that Wnt5a is expressed in the IRS, ORS, hair matrix and DP cells. E16.5, embryonic day 16.5; P8, postnatal day 8; DC, dermal condensation; DP, dermal papilla; HG, hair germ; IRS, inner root sheath; ORS, outer root sheath. N=9.

Hair follicle develops multiple components at embryonic day 16.5 (E16.5), and acquires its complete structures at postnatal day 8 (P8) (Figure 1A-1B). At E16.5, immunofluorescence staining shows that Wnt5a is expressed in the mesenchymal components, particularly in the dermal condensation (DC) of the developing hair follicle (Figure 1C). When hair follicle enters full anagen at P8, immunohistochemistry staining shows that Wnt5a is expressed at a high level in the inner root sheath (IRS), and a lower level in the outer root sheath (ORS), dermal papilla (DP) and hair matrix (Figure 1D).

### *Wnt5a* is decreased in *Gsdma3*-mutant mice in anagen phase

**Figure 2:**
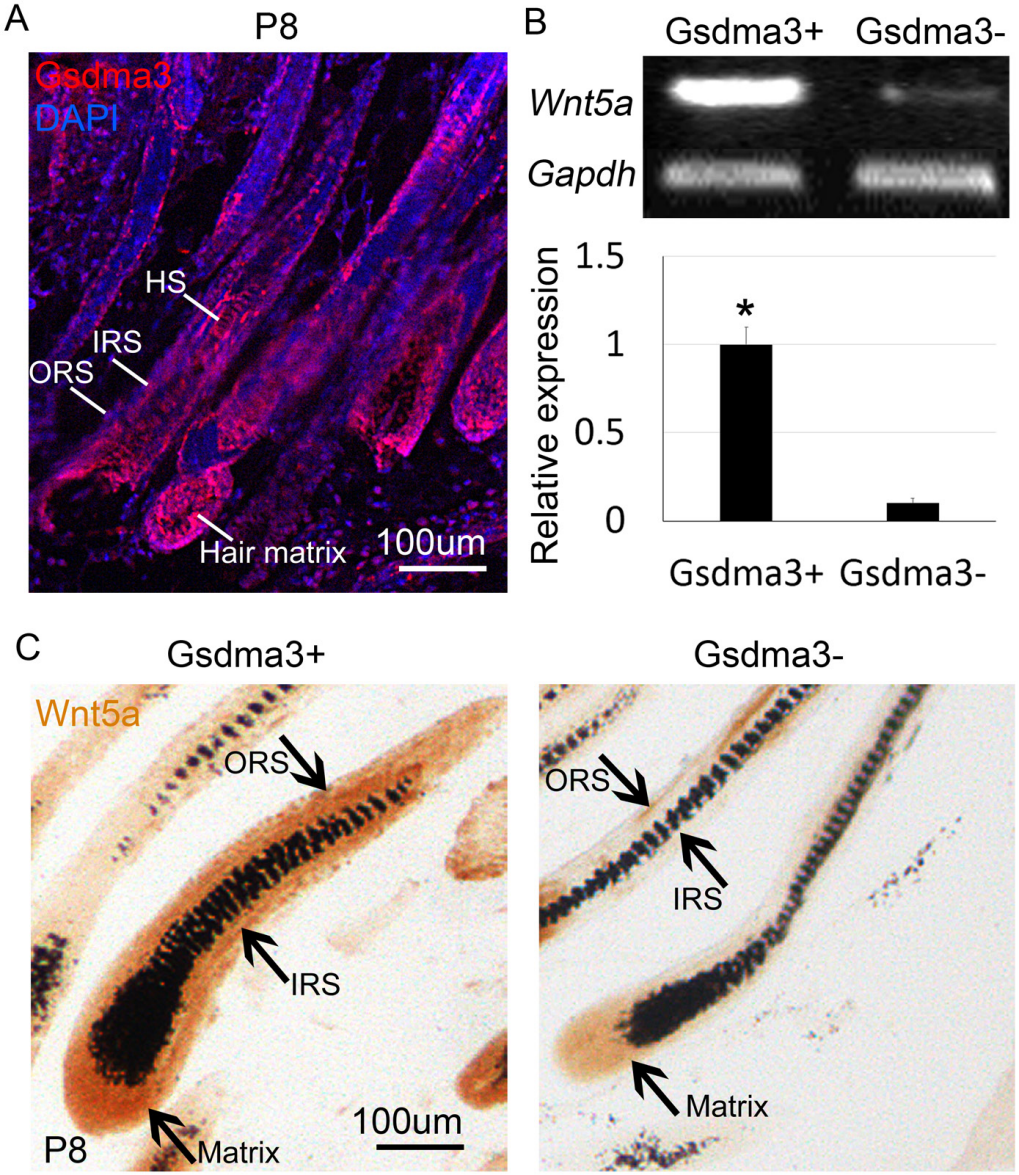
Decreased Wnt5a expression in *Gsdma3*-mutant mice **(A)** Immunofluorescent staining shows that *Gsdma3* is expressed in the IRS, ORS, hair shaft and hair matrix of the hair follicle in mice. **(B)** RT-PCR and analysis reveal that mRNA of *Wnt5a* expression is significantly decreased in *Gsdma3*-mutant mice skin when compared to the wild type mice. **(C)** Immunohistochemistry staining shows that Wnt5a expression is decreased in *Gsdma3*-mutant mice at ORS, IRS and hair matrix region at mid-anagen phase. P8, postnatal day 8; IRS, inner root sheath; ORS, outer root sheath. N=9.

We recently found that *Gsdma3* is also expressed in the hair follicle [24]. With a similar expression pattern to *Wnt5a*, *Gsdma3* also shows an elevated expression in the IRS, and a reduced expression in ORS of the hair follicle (Figure 2A). Therefore, we speculated that there is an interaction between Gsdma3 and Wnt5a. To verify this, we harvested skin from WT mice and *Gsdma3*-mutant mice at the full anagen phase at P8. RT-PCR and its analysis show that the mRNA expression of Wnt5a is significantly decreased in the *Gsdma3*-mutant mice when compared to the WT mice (Figure 2B). Immunohistochemistry staining shows that Wnt5a is decreased in its expression to the IRS, ORS and hair matrix regions of the hair follicle in *Gsdma3*-mutant mice, compared to that of WT mice (Figure 2C). This suggests that the expression level of Wnt5a may be regulated by Gsdma3.

### Overexpression of *Gsdma3* promotes *Wnt5a* expression in epidermal cells

**Figure 3:**
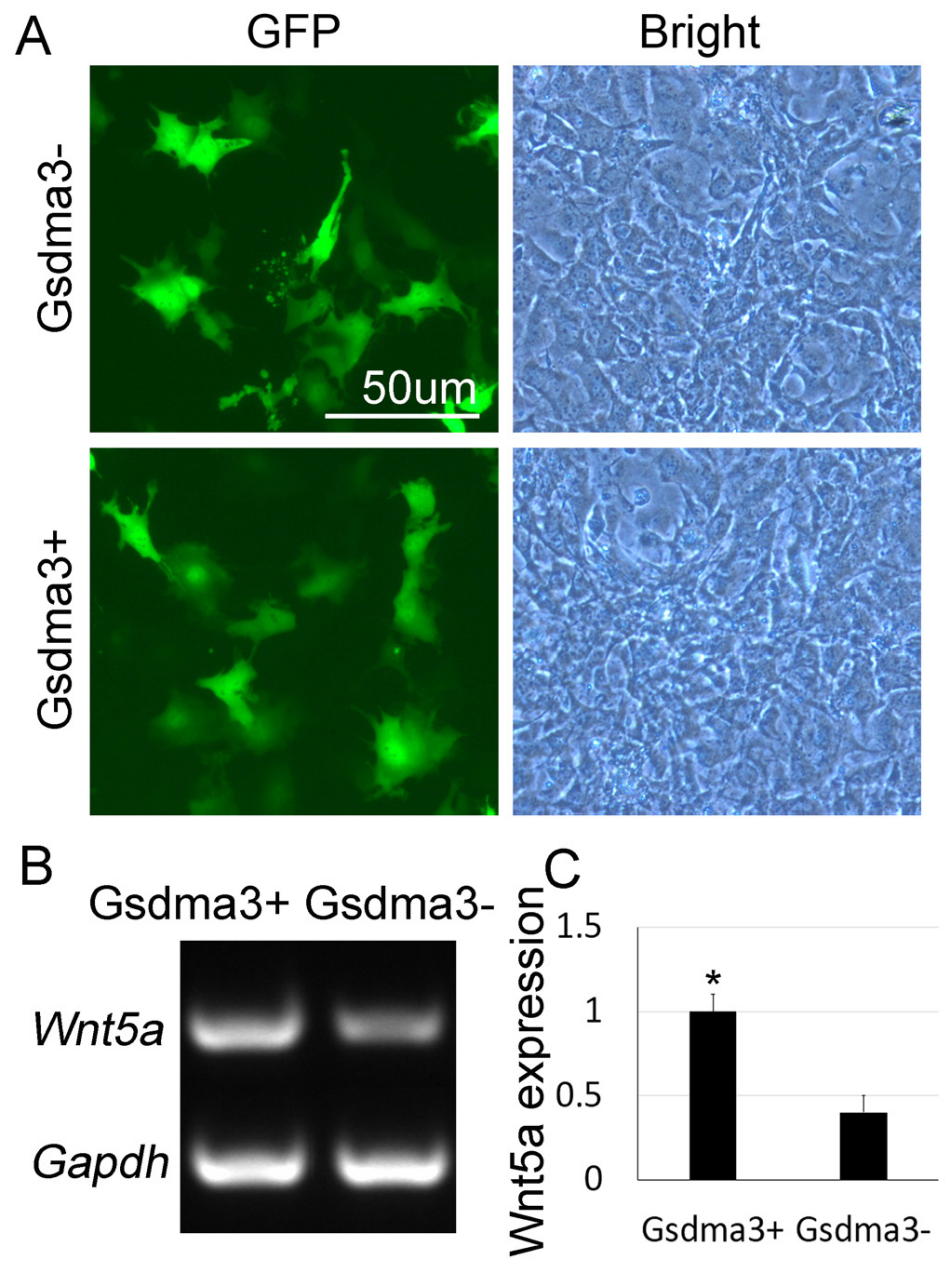
Overexpression of *Gsdma3* up-regulates *Wnt5a* mRNA expression in epidermal cells **(A)** Green fluorescence indicates successful transfection of *Gsdma3* overexpression plasmid and control plasmid. **(B)** RT-PCR shows mRNA expression *Wnt5a* after *Gsdma3* overexpression in epidermal cells. **(C)** Statistical analysis reveals that *Wnt5a* mRNA expression is significantly increased after in *Gsdma3* overexpression group, compared to the control group. Gsdma3+, Gsdma3 overexpression group; Gsdma3-, control group. ^*^P<0.5. N=9.

To examine if Gsdma3 regulates *Wnt5a* in epithelial cells, we constructed *Gsdma3* overexpression plasmids and transfected them into the cultured JB6 cells. Green fluorescence shows successful transfection of *Gsdma3*-expressing plasmid (Gsdma3+) and the control plasmid (Gsdma3-) into JB6 cells 24h after culture (Figure 3A). At this time point, RT-PCR and its analysis show that *Wnt5a* expression is significantly increased in *Gsdma3*-overexpressed JB6 cells, when compared to the control group (Figure 3B-3C). These results further suggest that Gsdma3 may regulate and maintain *Wnt5a* expression levels within a certain range.

### Overexpression of *Wnt5a* suppresses Wnt signaling in DP cells

**Figure 4:**
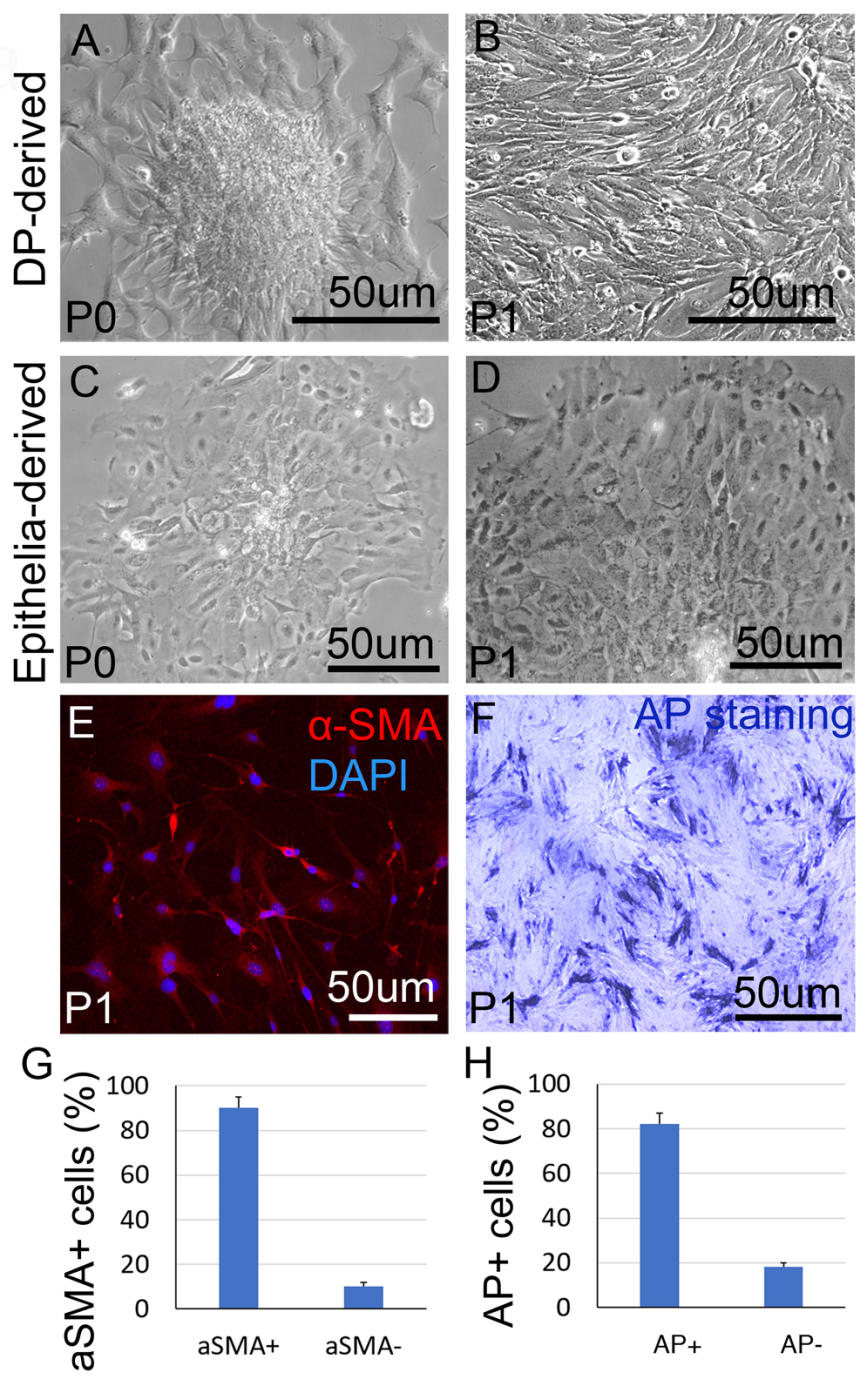
DP cell culture **(A)** A DP from mouse whisker is expanded during culture. **(B)** DP cells grow in a spindle shape after 1 passage culture. **(C)** Primary epithelial cell culture from mouse hair follicle. **(D)** Epithelial cells maintain their morphology after 1 passage culture. **(E)** Immunostaining shows positive a-SMA expression in cultured DP cells. **(F)** DP cells show positive alkaline phosphatase staining after 1 passage culture. **(G)** Statistical analysis reveals that more than 80% P1 cells are aSMA positive; **(H)** Statistical analysis reveals that about 80% P1 cells are alkaline phosphatase (AP) positive; P1, subculture for 1 passage. N=9.

Recent study shows that the formation of seven concentric differentiating epithelial layers of the hair follicle is the result of epithelial-DP interaction [28]. While *Gsdma3* is expressed exclusively in epithelial cells [29]. To verify how epithelia-expressed *Gsdma3* regulates *Wnt5a* expression to impact DP cells, we first isolated DP cells from the mouse whisker. DP-derived cells from the whisker show a different shape, compared to the epithelial-derived cells from the bulge of the hair follicle. When passaged for 1 time (P1), DP-derived cells display spindle shape with more filopodia (Figure 4A-4B), whereas epithelial-derived cells show an oval shape with more lamellipodia (Figure 4C-4D). 80% the P1 cells show aSMA-positive and alkaline phosphatase (AP)-positive, indicating at least several cells have maintained the DP characteristics during culture (Figure 4E-4H).

**Figure 5:**
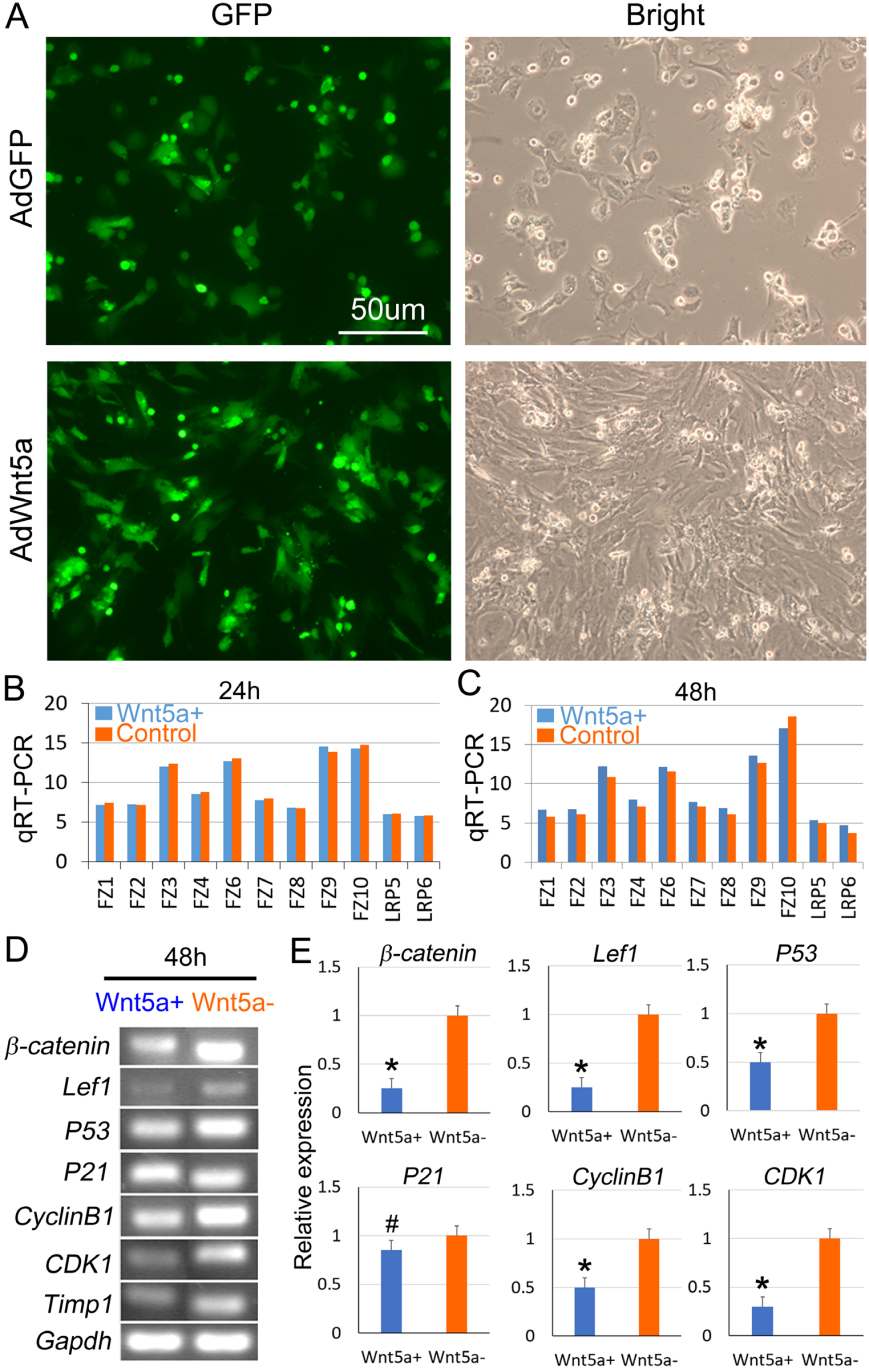
Overexpression of Wnt5a attenuates canonical Wnt signaling pathway in cultured DP cells **(A)** Green fluorescence indicates successful infection of AdWnt5a and AdGFP control adenoviruses. **(B)** qRT-PCR shows that no significant change of Frizzled receptors expression in cultured DP cells 24h after Wnt5a treatment, compared to the control group. Blue bars represent Wnt5a overexpression groups, and deep yellow bars represent control groups. **(C)** qRT-PCR shows that no significant change of Frizzled receptors expression in cultured DP cells 48h after Wnt5a treatment, compared to the control group. **(D)** RT-PCR shows that expression of genes involved in Wnt pathway and cell cycling in cultured DP cells 48h after Wnt5a treatment, compared to the control group. **(E)** Statistical analysis reveals differential expression of genes involved in Wnt pathway and cell cycling in cultured DP cells 48h after Wnt5a treatment, compared to the control group. P<0.5. N=3.

We then overexpressed *Wnt5a* by applying *Wnt5a*-expressing adenovirus (AdWnt5a) into the P1 DP cells. Green fluorescence shows successful infection of AdWnt5a and the control adenovirus (AdGFP) into DP cells 24h after culture (Figure 5A). However, real-time PCR results show that the mRNA expression of Wnt pathway receptors including Fzds 1, 2, 3, 4, 6, 7, 8, 9, and 10, Lrps 5 and 6 are not significantly changed in the AdWnt5a-treated group, compared to the AdGFP-treated group 24h and 48h after culture (Figure 5B-5C). This indicates that Wnt5a cannot increase the expression of frizzled receptors in cultured DP cells. Intriguingly, RT-PCR and its analysis show that the expression of key genes in canonical Wnt signaling pathway such as β-catenin and Lef1 is significantly decreased in DP cells after AdWnt5a treatment, when compared to the control group (Figure 5D-5E).

*P53* gene has been reported to play an important role in cell cycle regulation [30]. *P21* is a downstream target which acts as a regulator of cell cycle progression at G1 and S phase [31]. Overexpression of *Wnt5a* significantly decreased *P53* expression but didn’t influence *P21* expression (Figure 5D-5E). In addition, overexpression of *Wnt5a* results in significantly decreased expression of cell cycle genes such as *CyclinB1* and *CDK1* expression in DP cells, when compared to the AdGFP control group (Figure 5D-5E).

## DISCUSSION

**Figure 6:**
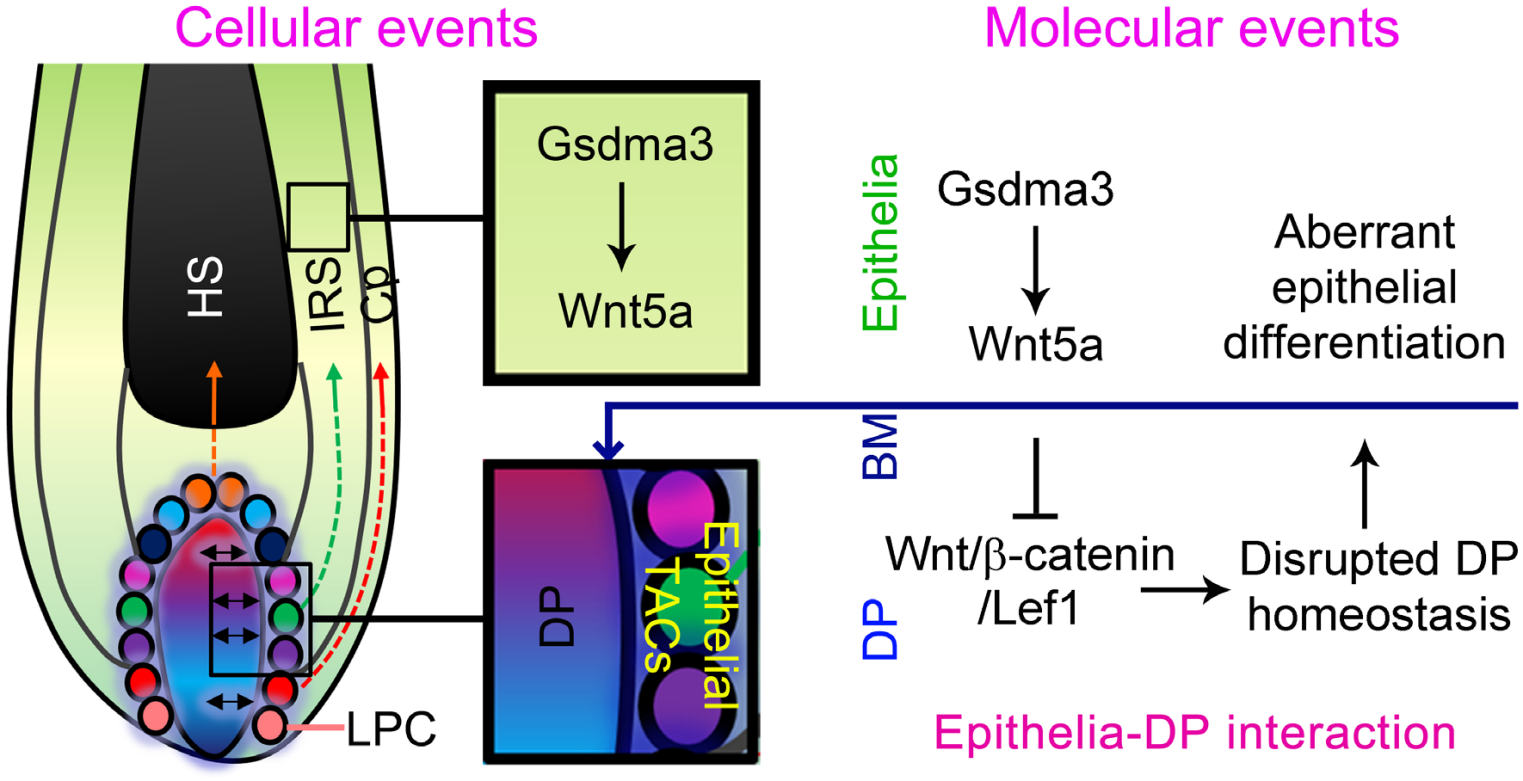
Epithelial-mesenchymal mechanism involved in hair follicle differentiation Normal expression of *Gsdma3* results in a proper hair follicle differentiation. In *Gsdma3*-mutant mice, loss of function of *Gsdma3* leads to decreased *Wnt5a* expression in epithelia of the hair follicle. Decreased *Wnt5a* in epithelia causes up-regulated canonical Wnt signaling pathway, leading to a disrupted homeostasis of the DP, which results in an aberrant epithelial differentiation. Whereas overexpression of *Wnt5a* results in decreased expression of canonical Wnt signaling pathway genes, which also leads to a disrupted homeostasis of the DP, in turn causing an aberrant epithelial differentiation. Colorful depicts represent the heterogeneity in TACs. BM, basement membrane; DP, dermal papilla; LPC, lower proximal cup; HS, hair shaft; IRS, inner root sheath; Cp, companion layer; TACs, transient amplifying cells.

Increasing evidence shows that non-canonical Wnt signaling pathway is critical for hair follicle development, growth and regeneration. As a key non-canonical Wnt pathway ligand, *Wnt5a* functions as downstream of Sonic Hedgehog pathway to regulate hair follicle morphogenesis [18]. Wnt5a inhibits hair shaft growth during organ culture [22], and prevents hair follicles entering from telogen to anagen phase during hair regeneration through inhibiting canonical Wnt signaling pathway [13, 20, 21]. We previously proposed that an activator-inhibitor mechanism that regulates hair regeneration during aging [4]. In this study, we show that *Wnt5a* may act as a downstream of *Gsdma3* to control hair follicle differentiation through an epithelial-mesenchymal interaction mechanism (Figure 6). Specifically, we demonstrate that *Gsdma3* promotes *Wnt5a*-mediated non-canonical Wnt signaling pathway to orchestrate hair follicle differentiation.

Our group has uncovered that *Gsdma3* is expressed in the IRS and hair shaft of the hair follicle [24], and is required for hair follicle differentiation [27]. *Wnt5a* which has the similar expression pattern with *Gsdma3*, is down-regulated in the *Gsdma3*-mutant mice, suggesting that *Gsdma3* may positively regulate *Wnt5a* expression in the differentiated layers of the hair follicle. There are at least two possible mechanisms that *Gsdma3* regulates *Wnt5a* to influence hair differentiation. First, the intrinsic mechanism. Our data show that both *Gsdma3* and *Wnt5a* are expressed in the epithelial cells including in the hair shaft and IRS of the hair follicle, and overexpression of *Gsdma3* causes *Wnt5a* up-regulation in skin epidermal cells. It is possible that up-regulated *Wnt5a* leads to the differentiation of the local epithelial cells in the IRS and hair matrix. Given this is true, local epithelia-derived *Wnt5a* should directly promote hair follicle differentiation. However, overexpression of *Wnt5a* in the hair follicle epithelia results in a shortened hair shaft [22] and a delayed hair regeneration [13], indicating epithelial *Wnt5a* may prevent rather than promoting hair follicle differentiation. Thus, we tend to speculate that the second mechanism that *Wnt5a* regulates hair follicle differentiation through the epithelial-mesenchymal interaction.

Epithelial-mesenchymal interactions are essential for hair follicle development, regeneration and function. Complex structures of the hair follicle form as a result of sequential, coordinated and reciprocal interaction between epithelium and mesenchyme. Recent studies showed that there are four molecularly distinct cell populations located in the dermal papilla [28]. They constitute micro-niches with the juxtaposed epithelial transient amplifying cells to govern the differentiation of keratinocytes into the seven concentric differentiating layers of the hair follicle. Transcriptome analysis revealed that *Wnt5a* is not only expressed in the DP, but also a signature of the cortex of the hair shaft. Indeed, *Wnt10b* as one of the ligands mediating canonical Wnt pathway, is expressed in the transient amplifying cells that give rise to the IRS. Whereas nuclear Lef1 as the proxy of Wnt signaling, is expressed in both IRS lineages and mid-DP. The interaction between epithelial and DP regulates IRS differentiation during hair growth. While *Wnt5a* is expressed both in epithelial and DP, *Gsdma3* is specifically expressed in the epithelial tissues [29]. Thus, similar to *Wnt10b*, it is tempting to speculate that *Gsdma3* promotes *Wnt5a* expression in the epithelia, then epithelial-*Wnt5a* influences DP cells which in turn regulate differentiation of epithelial transient amplifying cell through epithelial-DP interaction.

Wnt5a functions as a downstream mediator of Notch/CSL signaling in the DP to regulate the hair follicle differentiation [23]. How does *Wnt5a* influence DP cells to regulate epithelial cell differentiation? *Wnt5a* is able to attenuates Wnt/β-catenin signaling in cultured human DP cells [32], and suppresses Wnt/β-catenin signaling during telogen-to-anagen transition. Our results also showed that overexpression of Wnt5a in DP cells results in decreased expression of canonical Wnt pathway genes such as β-catenin and Lef1. These studies suggest that *Wnt5a* is sufficient to inhibit canonical Wnt signaling pathway both *in vivo* and *in vitro* in mouse cells. Therefore, canonical Wnt signaling pathway genes are significantly increased [12, 24] in *Gsdma3*-mutant mice in which *Wnt5a* expression is decreased. Canonical Wnt signaling is important for regulating hair follicle differentiation. Thus, decreased *Wnt5a* expression results an aberrant hair shaft differentiation in the hair follicle.

In addition, it is obvious that the homeostasis of the DP is changed. Our PCR results show that *P53* expression is significantly decreased in cultured DP cells after *Wnt5a* treatment. *P53* has various functions such as regulating DNA repair, cell aging, senescence and apoptosis. *P53* binds to *P21* to establish a longer G1 phase during cell cycle [33]. However, in our study, we found that *P21* is not changed in DP cells after *Wnt5a* treatment, suggesting that *P53* may not promote DP cell apoptosis *in vitro*. Indeed, we also didn’t observe significant cell apoptosis in the DP cells in *Gsdma3*-mutant mice *in vivo* [25, 26]. On the other side, other cell cycle genes such as *Cyclin B1* and *CDK1* that are involved in G2/M phase are also decreased in DP cells after *Wnt5a* treatment, indicating *Wnt5a* may prevent DP cell proliferation. Previous study shows that *Wnt5a* suppresses hematopoietic stem cells by maintaining them in a quiescent G0 state [16]. *P53* also plays important role in promoting embryonic stem cells differentiation [34]. Thus, decreased expression *P53* in the DP cells after *Wnt5a* treatment may function to regulate maintaining DP cells in a more quiescent state. However, active cellular behaviors occur during hair follicle structure reestablishment during anagen phase, relying largely on epithelial-DP interaction. The relatively quiescent DP cells may also contribute to aberrant hair follicle differentiation in *Gsdma3*-mutant mice.

Taking together, our findings demonstrate that *Gsdma3* promotes hair follicle differentiation by positively regulating *Wnt5a* expression in the epithelia of the hair follicle. Epithelia-derived *Wnt5a* further influences DP cells which govern the adjacent epithelial transient amplifying cells to differentiate into the hair shaft and IRS (Figure 6). How the molecular change of DP cells influences DP-epithelial interaction remains further investigation. Our study provides new clues not only for the relationship between canonical and non-canonical Wnt signaling pathways in regulating hair follicle growth, but also enhances the mechanism of epithelial-mesenchymal interactions in governing hair follicle differentiation.

## MATERIALS AND METHODS

### Mice

*Gsdma3*-mutant mice were gifted from National Database of Mouse Genetic Resources, Nanjing, China. The mutant mice were generated from a point mutation (nt1112 T to C) on a C57BL/6 background by ENU (ethylnitrosourea). Both *Gsdma3*-mutant mice and C57BL/6 wild mice were housed at the Experimental Animal Center of the Third Military Medical University, China. All the experimental protocols were approved by the Research Committee of the Third Military Medical University.

### Histology and immunostaining

Skin samples were harvested from the dorsal track of *Gsdma3*-mutant mice or C57BL/6 mice, and fixed in 4% paraformaldehyde in PBS for overnight. Sections were cut at 5 mm, dewaxed by xylene, and rehydrated by ethanol in gradient concentrations. For hematoxylin-eosin (H&E) staining, sections were stained with hematoxylin and eosin for 1 min, respectively, and then mounted with resinene. For immunostaining, sections were subjected to antigen retrieval with 0.1M citric acid buffer at ph6.0. Then the samples were incubated with primary antibodies against aSMA (1:100, Boisynthesis Biotechnology Co., Ltd. Beijing, China), Wnt5a (1:1000, Abcam, Cambridge, USA) and Gsdma3 (1:100, GL Biochem, Shanghai, China) [24] at 4°C for overnight. After wash, the samples were incubated with Alexa Fluor 594 (1:500, Beyotime, Jiang Su, China) conjugated secondary antibodies. Nucleus was labeled with 4’, 6’-diamidino-2-phenylindole (DAPI, 1: 1,000, Sigma-Aldrich, St. Louis, MO, USA).

### Reverse transcription polymerase chain reaction (RT-PCR)

Total RNA was extracted by using Trizol reagent (Life Technologies, Grand Island, USA). One ug of total RNA was reversely transcribed to cDNA by using a RT kit (Toyobo, Japan), at 30°C for 10 min, 42°C for 20 min, 94°C for 10 min, and 4°C for 5 min in order. The primers used in the PCR reactions were listed in Supplementary Table 1. The TM value for PCR was set at 63°C.

### Plasmid and adenovirus

Generation of *Gsdma3* overexpression plasmid was as previously described [26]. In brief, CDS sequence of the *Gsdma3* gene was cloned into a pEGFP-N1 plasmid, with a forward primer 5’-ACGCGTCGACATGCCTGTGTTTGAGGATGTCAC-3’ and a reverse primer 5’-TCCCCGCGG AGATAGAGCACAATAAGTAAGTGCATTAGA-3’. Adenovirus-mediated overexpression of *Wnt5a* (AdWnt5a) and AdGFP (control) adenoviruses were gifted from Dr. Tong-Chuan He (University of Chicago, USA). Adenoviruses were propagated in HEK293 cells.

### Cell culture and infection

For culture of hair follicle epithelial cells, vibrissa was dissected out by using a micro-needle from postnatal day 8 (P8) mice. After wash in PBS, the vibrissa was placed into a culture dish, and cultured in DMEM culture medium containing 10% fetal bovine serum (FBS).

JB6 Cl 30-7b (JB6 cells) mouse epithelial cell line was purchased (ATCC, Manassas, USA). JB6 cells were cultured in DMEM culture medium containing 10% FBS. Four ug mouse recombinant *Gsdma3* overexpression plasmid or pEGFP-N1 control plasmid was transfected to JB6 cells by using a lipofectamine 2000 kit (Life Technologies, Grand Island, USA). Cells were harvested for RNA extraction 48 hours after plasmid transfection.

For culture of DP cells, vibrissa was dissected out from P8 mice. After wash in Hanks solution, the vibrissa was treated with 0.25% dispase at 37°C for 40 min. Then the DP was dissected out by using a micro needle, digested by 0.25% dispase at 37°C for 1.5 hours, and then centrifuged at 500 rpm for 3 min. The cells were re-suspended and digested again by 0.25% dispase at 37°C for 30 min. After being centrifuged at 2000 rpm for 3 min, the cells were seeded into a culture dish with DMEM culture medium containing 10% FBS.

### Alkaline phosphatase staining

DP cells were passaged into a 24-well culture plate placed with small cover glasses. 24 hours after culture, the cells adhered to the cover glasses were washed in PBS for 3 times, and incubated with NBT/BCIP solution at room temperature for 10 min.

### Statistical analysis

All experiments were repeated at least three times. The statistical analysis was expressed at mean ± SD. Student’s t test was used to evaluate the statistical significance, which was set as p < 0.05.

## SUPPLEMENTARY MATERIALS TABLE



## Abbreviations

Ad: adenovirus
DP: dermal papilla
IRS: inner root sheath
ORS: outer root sheath
TACs: transient amplifying cells
Wnt: wingless-type MMTV integration site family
