# Supplementary material for: Gsdma3 regulates hair follicle differentiation via Wnt5a-mediated non-canonical Wnt signaling pathway
Source: Oncotarget. 2017 Oct 31;8(59):100269–79. doi: 10.18632/oncotarget.22212 (PMC5725018; doi:10.18632/oncotarget.22212)
Supplement: Supplementary file 1 [file oncotarget-08-100269-s001.pdf]

Gsdma3 regulates hair follicle differentiation via Wnt5a-mediated non-canonical Wnt signaling pathway

SUPPLEMENTARY MATERIALS

Supplementary Table 1: Primers used for PCR

Gene name	Primer sequences (5'-3' orientation)
Wnt5a Fwd	CTCCTTCGCCCAGGTTGTTATAG
Wnt5a Fwd	GGTCCTGATACAAGTGGCAGAGT
Gapdh Fwd	GACATCAAGAAGGTGGTGAAGC
Gapdh Rev	GAAGGTGGAAGAGTGGGAGTT
Frizzled1 Fwd	ACTATAACCATCTTGGCGTTGG
Frizzled1 Rev	TGAACAGATAAACGAAGAGAGGC
Frizzled2 Fwd	GCCGTCCTATCTCAGCTATAAGT
Frizzled2 Rev	TCTCCTCTTGCGAGAAGAACATA
Frizzled3 Fwd	CGTCACAAGATTCCGTTACCC
Frizzled3 Rev	CTACTCGGTCCTCCAGCAAAA
Frizzled4 Fwd	GCCCCACAAGACTCCCATC
Frizzled4 Rev	CCAGCATCGTAGCCACACT
Frizzled6 Fwd	TCTTCCCTAACCTGATGGGTC
Frizzled6 Rev	ACAATTTCCGACAGGGTAGAAC
Frizzled7 Fwd	AGACCCACCTTTCACTGCG
Frizzled7 Rev	AAGTACATGAGGCCGTTAGCA
Frizzled8 Fwd	GGGTTACCTGTTGGAAGTGAC
Frizzled8 Rev	GGCACCGTGATCTCTTGGC
Frizzled9 Fwd	TTGCTCTATTATTTCGGGATGGC
Frizzled9 Rev	CAGGACCACGATAGTTTTGAGTG
Frizzled10 Fwd	GCAAGCTCCCCAACAAGAAC
Frizzled10 Rev	CCCGTCCTTTAGTGGGTGC
LRP5 Fwd	CGCAGGGCGTACCTAGATG
LRP5 Rev	CGAGTCACCTCAATTCTGTCAG
LRP6 Fwd	TTGTTGCTTTATGCAAACAGACG
LRP6 Rev	GTTCGTTTAATGGCTTCTTCGC
β- catenin Fwd	ATGGAGCCGGACAGAAAAGC
β- catenin Rev	CTTGCCACTCAGGGAAGGA
Lef1 Fwd	AACGAGTCCGAAATCATCCCA
Lef1 Rev	GCCAGAGTAACTGGAGTAGGA
P53 Fwd	ATTTGTATCCCGAGTATCTG
P53 Rev	GGTATACTCAGAGCCGGCCT
P21 Fwd	AATGGAGACAGAGACCCCAGA
P21 Rev	TAAGGGCCCTACCGTCCTAC
CyclinB1 Fwd	TCTCGAATCGGGGAACCTCT
CyclinB1 Rev	TCGGGCTTGGAGAGGGATTA
CDK1 Fwd	ACAGAGAGGGTCCGTCGTAA
CDK1 Rev	AAAGTACGGGTGCTTCAGGG
Timp1 Fwd	TACCATGATGGCCCCCTTTG
Timp1 Rev	ACTCTTCACTGCGGTTCTGG

Fwd, forward primer; Rev, reverse primer.
